# Zinc gluconate protects against plant virus infection in tomato and *Nicotiana benthamiana* plants

**DOI:** 10.5511/plantbiotechnology.24.0628a

**Published:** 2024-12-25

**Authors:** Mari Narusaka, Yoshihiro Narusaka

**Affiliations:** 1 Okayama Prefectural Technology Center for Agriculture, Forestry, and Fisheries, Research Institute for Biological Sciences, Okayama 716-1241, Japan

**Keywords:** micronutrient, plant virus disease control, tomato mosaic virus, zinc gluconate

## Abstract

Plant viruses cause significant damage to global crop protection, since they can reduce plant quality and quantity, and the estimated annual cost of virus-induced damage is approximately $30 billion. Tomato mosaic virus (ToMV), a member of the *Tobamovirus* genus, presents a major threat to tomatoes and other solanaceous plants. Agricultural chemicals, including plant growth regulators, are commonly used to control the spread of pathogens, but these can be ineffective against viruses. In this study, we aimed to develop an antiviral agent using micronutrients such as zinc, iron, and copper. The plant virus disease control effects of these micronutrients was evaluated by applying zinc gluconate (ZnGluc), iron gluconate (FeGluc), and copper gluconate (CuGluc) solutions to *Nicotiana benthamiana* plants that were subsequently inoculated with ToMV. Our results showed that ZnGluc exhibited the highest disease control activity and did not cause phytotoxic effects. Further analysis via quantitative real-time polymerase chain reaction analysis confirmed these findings. In addition, a mixture of ZnGluc and proanthocyanidins sourced from *Alpinia zerumbet* extracts exerted a synergistic disease control effect. Overall, we provide the first evidence that micronutrients, especially ZnGluc, exhibit significant disease control activity against ToMV, and thereby suggest that these treatments have potential as an agricultural chemical.

Plant viruses cause significant damage to agricultural and horticultural crops throughout the world. Depending on the host-virus pair, infected plants can show various symptoms, including mosaic patterns, stunting, yellowing, leaf curling, and necrosis, all of which can lower crop quality and quantity. The global cost of damage caused by plant viruses is estimated at approximately $30 billion per year (Jones and Naidu 2019).

Tomato mosaic virus (ToMV) belongs to the genus *Tobamovirus* in the family *Virgaviridae*. ToMV has a wide range of hosts, including solanaceous species such as tomato (*Solanum lycopersicum* L.), pepper (*Capsicum annuum* L.), and *Nicotiana benthamiana* Domin. ToMV is critical pathogen of global tomato crops, and causes significant reduction in tomato quantity and quality. Agricultural chemicals are typically used to control pathogen spread, although they cannot be used to directly control the spread of plant virus diseases. Therefore, in this study we aimed to develop an antiviral agent using micronutrients. Various micronutrients are known to enhance plant health and immunity and may therefore potentially increase resistance to viral infection (Tripathi et al. 2022). For example, micronutrients such as zinc, iron, and copper play important roles for plant growth and health. Despite evidence that micronutrients can strengthen plant immune systems and increase resistance to pathogens and various stressors, it remains unclear whether they also inhibit plant viruses and/or can effectively treat infections. Consequently, the suitability of their use as agricultural chemicals has yet to be evaluated (Tripathi et al. 2022).

In this study, we examined the effect of three micronutrients: zinc, iron, and copper on viral infection. To do so, we applied foliar sprays containing zinc gluconate (ZnGluc), zinc sulfate, iron gluconate (FeGluc), and copper gluconate (CuGluc) to *N. benthamiana* plants at the third true leaf stage. After a single foliar application, plants were inoculated with ToMV three days posttreatment. Inoculation of *N. benthamiana* plants with ToMV was performed as described in a previous study (Narusaka et al. 2020), with some modifications as follows. Briefly, we used pTL-derived plasmids (pTLBN. G3), which contain a full-length ToMV complementary DNA (cDNA) and a gene encoding green fluorescent protein (GFP) (Kubota et al. 2003). We performed in vitro transcription using 2 µg samples of this template DNA using the AmpliCap-Max T7 High Yield Message Maker Kit (CELLSCRIPT, Madison, WI, USA). This reaction was performed at 37°C for 40 min in a 20 µl reaction volume, and the resulting transcription mixture was subsequently diluted 40-fold in water. Furthermore, 20 µl of the diluted mixture was mixed with abrasive carborundum (600 mesh; Nacalai Tesque, Kyoto, Japan) before being mechanically applied to the third true leaves of *N. benthamiana*. Here, plants were either treated with a sample solution or a distilled water control. GFP foci were then used to detect virus infection, and they were observed under blue-light irradiation three days post-inoculation (dpi).

Overall, our results showed that treatment with ZnGluc, FeGluc, and CuGluc effectively protected *N. benthamiana* leaves against ToMV-GFP infection relative to a water-treated control (Figure 1A, B). In addition, application of 20 mM ZnGluc showed greater disease control activity than treatment with FeGluc or CuGluc, but exerted no clear phytotoxic effects related to plant growth inhibition, yield reduction, or leaf burn. In contrast, we observed chemical damage to plants when zinc sulfate, FeGluc, and CuGluc were used at comparable concentrations (Supplementary Figure S1). Next, we used quantitative real-time polymerase chain reaction (qRT-PCR) analysis to validate these findings at the RNA level. The qRT-PCR analysis was conducted following the procedures outlined in the Supplementary Methods. Briefly, equal amounts of total RNA were subjected to cDNA synthesis before specific ToMV sequences were then amplified. qRT-PCR results (Figure 1C) did not significantly differ from those obtained from assays based on the number of GFP foci (Figure 1A, B). Overall, our results indicate that in *N. benthamiana* plants, treatment with ZnGluc, FeGluc, and CuGluc protected inoculated and uninoculated upper leaves against ToMV relative to water-treated plants (Figure 1C). Further tests in a tomato host showed that ZnGluc also protected both inoculated and uninoculated upper leaves against ToMV relative to a water-treated control (Figure 2).

**Figure 1:**
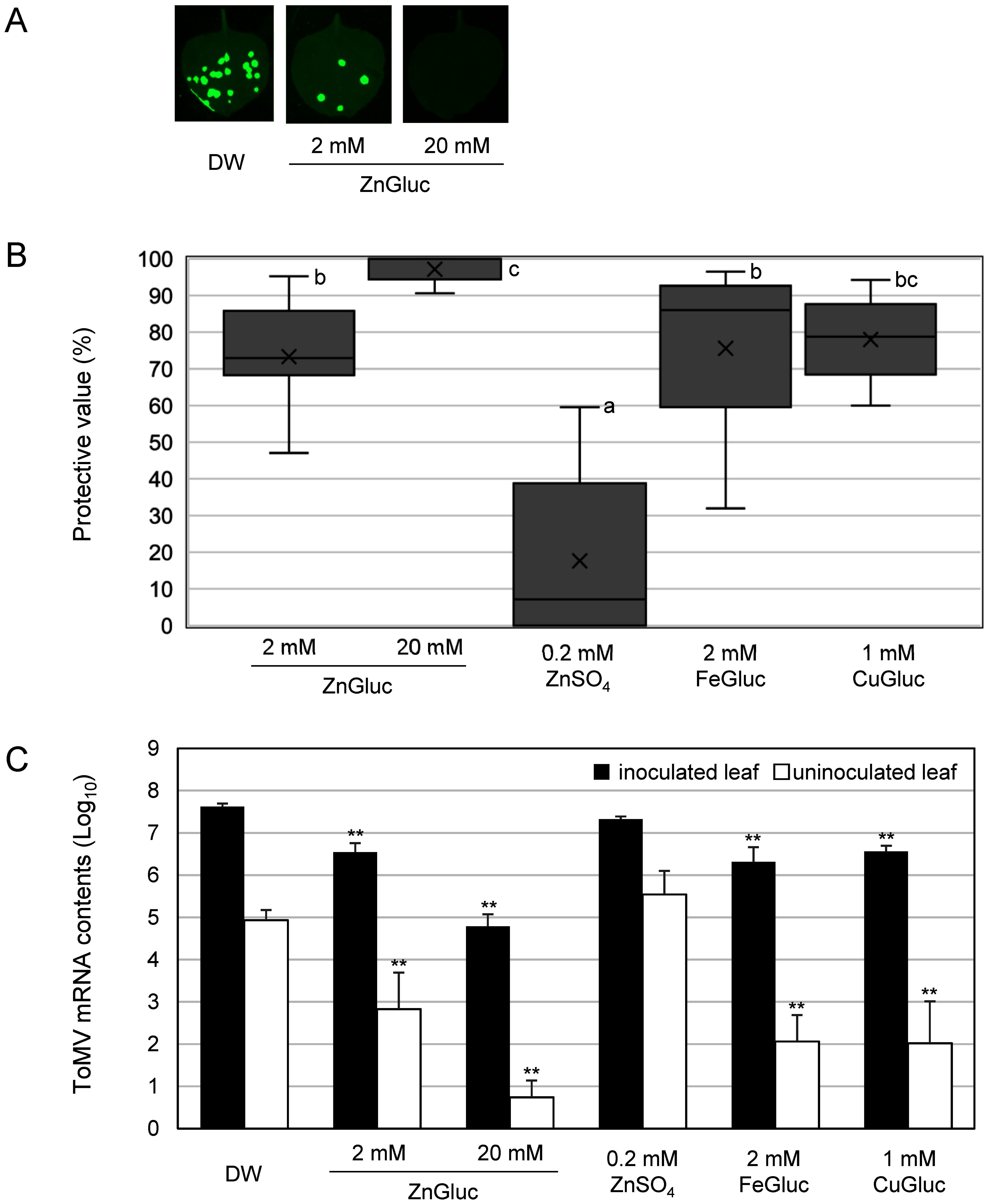
Figure 1. Effects of foliar application of micronutrients against GFP-tagged ToMV virions. *Nicotiana benthamiana* plants were treated with distilled water or micronutrient (ZnGluc, zinc sulfate, FeGluc, or CuGluc) foliar spray before being inoculated with GFP-tagged ToMV three days after treatment. (A) GFP foci on *N. benthamiana* at 3 dpi. Pictures were taken under blue-light irradiation with a ChemiDoc™ MP Imaging System (BIO-Rad Laboratories, Hercules, CA, USA). (B) Calculated number of GFP spots on the inoculated leaves at 3 dpi. Protective value=(1−number of GFP spots on micronutrient-treated plants/number of GFP spots on water-treated plants)×100. (C) ToMV mRNA levels in inoculated (3rd) and uninoculated upper (6th) leaves collected at 3 dpi. Bars indicate standard error (SE). The experiments (B, C) were independently conducted at least twice (*n*>3 per experiment) and the data from these experiments were combined. Different letters represent means that show statistically significant differences according to Tukey–Kramer multiple comparison tests (*p*<0.01). Asterisks indicate significant differences between pairs of control and treated plants at each leaf position as determined by Dunnett’s multiple comparison tests (** *p*<0.01).

**Figure 2:**
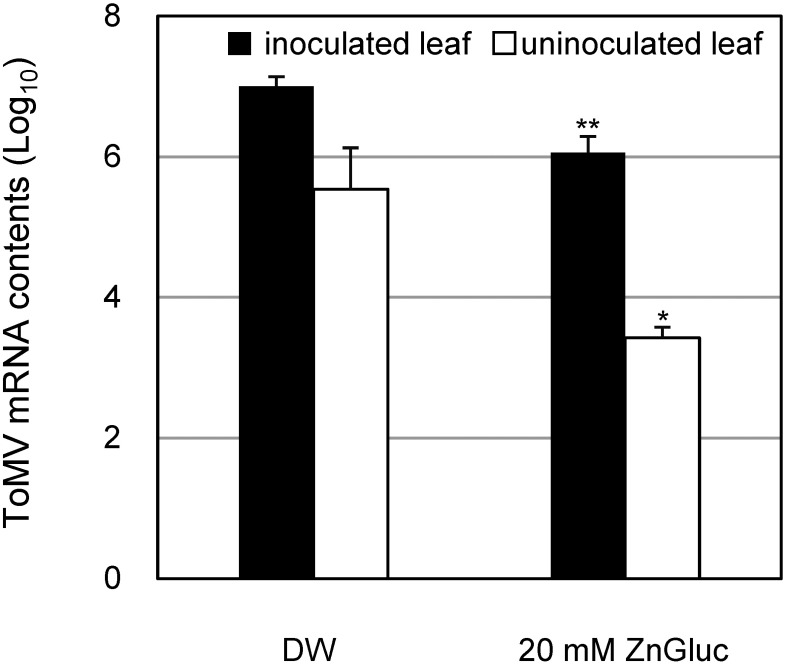
Figure 2. Disease control effects of ZnGluc in tomato plants following inoculation with GFP-tagged ToMV. Tomato plants (cv. Rejina, Sakata Seed Corp., Kanagawa, Japan) were treated with distilled water (DW) and 20 mM ZnGluc. Next, the first true leaf of tomato was inoculated with the GFP-tagged ToMV inoculum three days after treatment. Also shown are the levels of ToMV mRNA found in inoculated (1st) and uninoculated upper (4th) leaves collected at 6 dpi. Bars indicate standard error (SE). The experiment was independently conducted at least twice (*n*>3 per experiment) and the data from these experiments were combined. Asterisks indicate significant differences between pairs of control and treated plants at each leaf position as determined by Dunnett’s multiple comparison tests. (* *p*<0.05, ** *p*<0.01).

To mitigate the risk of developing chemical-resistant viruses due to the continuous use of a single type of agricultural chemical, the use of multiple chemical mixtures can be considered. We previously found that *Alpinia zerumbet* extracts contained proanthocyanidins (AzPACs), which show a high level disease control activity against ToMV infection in *N. benthamiana* (Hatanaka et al. 2021; Narusaka et al. 2020, 2021). Given the independent effectiveness of ZnGluc and AzPACs, we also tested their combined effect by assessing *N. benthamiana* plants treated with a mixture contain both ZnGluc and AzPACs against GFP-tagged ToMV. Encouragingly, these results showed that the mixture exerted a synergistic effect against disease control activity (Figure 3).

**Figure 3:**
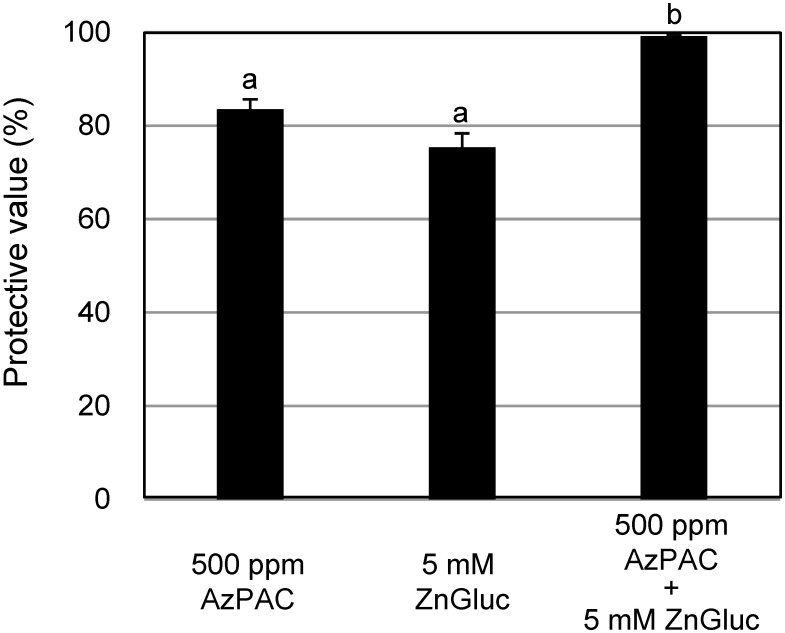
Figure 3. Effects of foliar application of a mixture of ZnGluc and *Alpinia zerumbet* extract containing proanthocyanidins (AzPACs) against GFP-tagged ToMV. *Nicotiana benthamiana* plants were treated with distilled water, ZnGluc, and/or AzPACs before being inoculated with GFP-tagged ToMV three days after treatment. The number of GFP spots on the inoculated leaves was calculated at 3 dpi. Bars indicate standard error (SE). The experiment was independently conducted at least twice (*n*>3 per experiment) and the data from these experiments were combined. Different letters represent means that show statistically significant differences according to Tukey–Kramer multiple comparison tests (*p*<0.01).

In this study, we demonstrate that micronutrients such as zinc, iron, and copper (especially ZnGluc) exert a high level of disease control activity against ToMV when used to treat infected tomato and *N. benthamiana* plants. In a previous report, foliar application of zinc sulfate was found to abate the symptoms of potato viruses (Ibrahim et al. 2016). In contrast, Deepika et al. (2021) identified no significant effect of single-agent treatment with zinc sulfate on the severity of papaya ringspot disease. In general, it is also known that excess levels of zinc cause phytotoxicity, and can inhibit plant growth and development, including leaf necrosis or chlorosis (Broadley et al. 2007). Therefore, chelation procedures that bind zinc to gluconic acid have reduced the levels of phytotoxicity observed when using inorganic zinc salts. Overall, our results indicate that ZnGluc has potential for further development as a novel control agent against plant viruses. Future research characterizing the disease control effects of ZnGluc may provide further insights into the use of control agents against plant viruses as agricultural chemicals.

## Abbreviations

AzPACs: *Alpinia zerumbet* extracts contained proanthocyanidins
CuGluc: copper gluconate
dpi: days post-inoculation
FeGluc: iron gluconate
GFP: green fluorescent protein
ToMV: tomato mosaic virus
ZnGluc: zinc gluconate

## Description of Supplementary Files

Supplementary Methods. Quantitative real-time PCR. SPAD value acquisition. Statistical analysis.

Supplementary Figure S1. SPAD value of *Nicotiana benthamiana* leaves treated with the micronutrients and/or proanthocyanidin.
