# Supplementary material for: Zinc gluconate protects against plant virus infection in tomato and Nicotiana benthamiana plants
Source: Plant Biotechnol (Tokyo). 2024 Dec 25;41(4):465–8. doi: 10.5511/plantbiotechnology.24.0628a (PMC11897722; doi:10.5511/plantbiotechnology.24.0628a)
Supplement: Supplementary Data [file plantbiotechnology-41-4-24.0628a-s001.pdf]

Supplementary Files

Supplementary Methods

Quantitative real-time PCR

Quantitative real-time PCR (qRT-PCR) was used to detect and quantify tomato mosaic virus (ToMV) in infected *Nicotiana benthamiana* and tomato plants. Briefly, total RNA was isolated from both inoculated leaves and uninoculated upper leaves of ToMV-infected plants using a Maxwell RSC simplyRNA Tissue kit (Promega, Madison, WI, USA). Subsequent synthesis of first-strand cDNA from total RNA for qRT-PCR was then performed using a PrimeScript™ RT kit (Takara Bio Inc., Otsu, Japan) using 500 ng of total RNA and oligo dT primers. Here, all procedures were performed according to the manufacturer's protocol.

qRT-PCR was performed using SsoAdvance SYBR Green Super mix (BIO-Rad Laboratories, Hercules, CA, USA) with a 1:10 diluted cDNA template and a CFX Connect Real-Time PCR Detection System (BIO-Rad Laboratories). qRT-PCR mixtures consisted of 1× SsoAdvance SYBR Green Super mix and 200 nM (each) of the sense and antisense primers. Following a preliminary denaturation step at 95°C for 3 min, reaction mixtures were then subjected to 40 cycles at 95°C for 5 s, and either at 63°C for 20 s (*N. benthamiana*), or 60°C for 20 s (tomato and ToMV). The target sample copy number was then averaged for two reactions. The experiment was independently conducted at least twice (n > 3 per experiment) and the data from these experiments were combined.

Next, expression of ToMV mRNA was used to estimate the level of ToMV infection. The nucleotide sequences of ToMV-specific primers for the target genome sequence are as follows: ToMV-forward primer 5′-GCGTTGGTAGTTACCTCAAG-3′, ToMV-reverse primer 5′-CTCCTCGCTCCTAGTGTATG-3′). The *NtEF1α* and *SLTIP41* genes were used for normalization in *N. benthamiana* and tomato plants, respectively. The nucleotide sequences of gene-specific primers for these genes are as

follows: *NtEF1α* (forward primer 5′-TCTGTTGAGATGCACCACGAAG-3′, reverse primer 5′-ACAAACCCACGCTTGAGATCC-3′); *SLTIP41* (forward primer 5′-ATGGAGTTTTTGAGTCTTCTGC-3′, reverse primer 5′-GCTGCGTTTCTGGCTTAGG -3′). qRT-PCR data characterizing the expression of ToMV mRNA, *NtEF1α* expression from *N. benthamiana*, and *SLTIP41* expression from tomato were expressed as log(copy number), a measure that was obtained from a standard curve of cycle times as a function of log(copy number). Next, the abundance of ToMV mRNA was normalized using *NtEF1α* or *SLTIP41* in infected samples. Subsequently, ToMV was quantified by calculating the number of copies of ToMV mRNA per *NtEF1α* or *SLTIP41* mRNA prepared from one infected *N. benthamiana* or tomato leaf, respectively.

SPAD value acquisition

Previous studies have demonstrated a positive correlation between the SPAD value and chlorophyll content (Ling et al. 2011). The change in the SPAD value can reflect the change in chlorophyll content (Yuan et al. 2016). In this study, we used the SPAD-502 meter (KONICA MINOLTA, INC., Tokyo, Japan) to directly measure the SPAD value of *N. benthamiana* leaves treated with distilled water (control), micronutrients and/or proanthocyanidin. The chlorophyll content of leaves, in which the leaf chloroplasts were damaged, gradually decreased, as did the SPAD value.

Statistical analysis

The statistical significance of observed differences in protective values between treated plants were analyzed using one-way analysis of variance (ANOVA) followed by Tukey–Kramer multiple comparisons test (Figure 1B, 3). Different letters represent statistically significant differences ($p < 0.01$). In addition, differences between control and treated plants at each leaf position were analyzed using ANOVA followed by

Dunnett's multiple comparisons tests (Figure 1C, 2). Differences with ★$p < 0.05$ or ★★$p < 0.01$ were considered to be statistically significant.

Supplementary Figure

Supplementary Figure S1. SPAD value of *Nicotiana benthamiana* leaves treated with the micronutrients and/or proanthocyanidin three days after treatment. The top and bottom black lines represent the maximum and minimum SPAD values, respectively. The cross represents the average SPAD value. Asterisks indicate significant differences between pairs of control and treated plants as determined by Dunnett's multiple comparison tests. (★★$p < 0.01$).

Supplementary References

Ling Q, Huang W, Jarvis P (2011) Use of a SPAD-502 meter to measure leaf chlorophyll concentration in *Arabidopsis thaliana*. *Photosynth Res* 107: 209-214

Yuan Z, Ata-Ul-Karim ST, Cao Q, Lu Z, Cao W, Zhu Y, Liu X (2016) Indicators for diagnosing nitrogen status of rice based on chlorophyll meter readings. *Field Crop Res* 185: 12-20

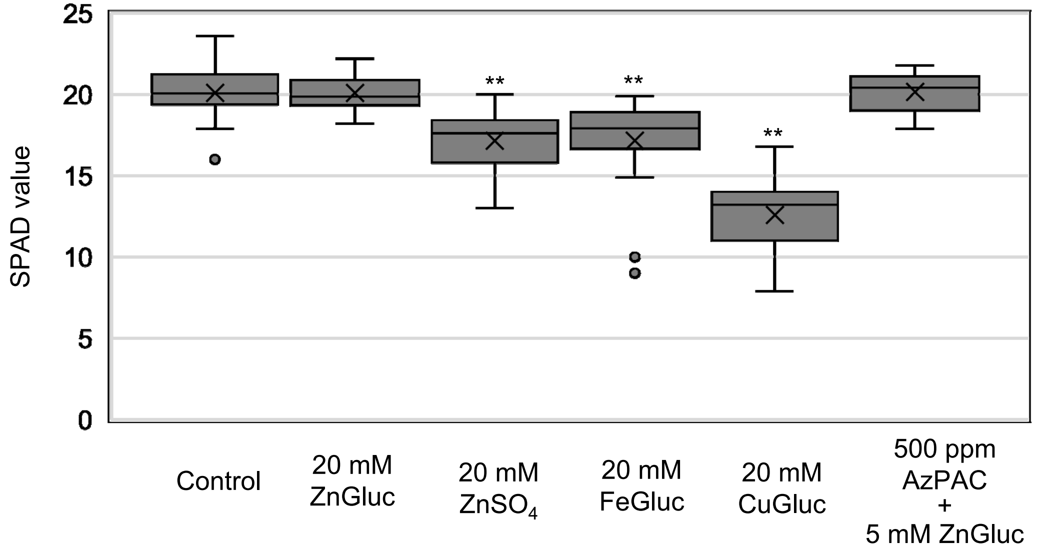

Suppl Figure S1 Narusaka et al.
